# Early Ileocecal Resection Is an Effective Therapy in Isolated Crohn’s Disease

**DOI:** 10.3390/jcm10040731

**Published:** 2021-02-12

**Authors:** Matthias Kelm, Friedrich Anger, Robin Eichlinger, Markus Brand, Mia Kim, Joachim Reibetanz, Katica Krajinovic, Christoph-Thomas Germer, Nicolas Schlegel, Sven Flemming

**Affiliations:** 1 Department of General, Visceral, Transplantation, Vascular and Pediatric Surgery, Center of Operative Medicine (ZOM), University Hospital of Wuerzburg, 97080 Wuerzburg, Germany; Kelm_M@ukw.de (M.K.); Anger_F@ukw.de (F.A.); Eichlinger_R@ukw.de (R.E.); Kim_M@ukw.de (M.K.); Reibetanz_J@ukw.de (J.R.); katica.krajinovic@klinikum-fuerth.de (K.K.); Germer_C@ukw.de (C.-T.G.); Schlegel_N@ukw.de (N.S.); 2 Department of Internal Medicine II, Section of Gastroenterology, Center of Internal Medicine (ZIM), University Hospital of Wuerzburg, 97080 Wuerzburg, Germany; Brand_M@ukw.de

**Keywords:** Crohn’s Disease, surgical therapy, ileocecal resection

## Abstract

Despite the increasing incidence and prevalence of Crohn’s Disease (CD), no curative options exist and treatment remains complex. While therapy has mainly focused on medical approaches in the past, growing evidence reveals that in cases of limited inflammation, surgery can suffice as an alternative primary treatment. We retrospectively assessed the disease course and outcomes of 103 patients with terminal Ileitis who underwent primary surgery (*n* = 29) or received primary medical treatment followed by surgery (*n* = 74). Primary endpoint was the need for immunosuppressive medication after surgical treatment (ileocecal resection, ICR) during a two-years follow-up. Rates for laparoscopic ICR were enhanced in case of early surgery, but no differences were seen for postoperative complications. In case of immunosuppressive medication, patients with ICR at an early state of disease needed significantly less anti-inflammatory medication during the two-year postoperative follow-up compared to patients who were primarily treated medically. Furthermore, in a subgroup analysis for patients with localized ileocecal disease manifestation, early surgery consistently resulted in a decreased amount of medical therapy postoperatively. In conclusion primary ICR is safe and effective in patients with limited CD, and the need for immunosuppressive medication during the postoperative follow-up is low compared to patients receiving surgery at a later stage of disease.

## 1. Introduction

Inflammatory Bowel Disease such as Crohn’s Disease (CD) represents a global socioeconomic burden throughout different health care systems [1,2]. Despite major efforts in basic and clinical research, its pathophysiology is still not understood in detail and no curative therapeutic options are currently available. Patients often have to take various medications with severe side effects which markedly reduce quality of life and significantly increase health care related costs [3,4,5]. Accordingly, introduction of new medical therapy biologicals initially demonstrated promising results with lower rates of surgical intervention, but long-term data showed otherwise [6,7]. Moreover, current studies demonstrate the positive effect of surgery with respect to increased quality of life and the decreased need for immunosuppressive medication in selected patient cohorts [8,9,10].

In cases of limited inflammation to the small bowel and/or colon, surgery in the form of ileocecal resection (ICR) might represent an alternative therapeutic option in CD compared to medical therapy due to its continued refinement and advances in minimal-invasive techniques [10,11,12]. Indeed, laparoscopic ileocecal resection in CD is not only a demonstrated safe surgical procedure, but also a cost-effective treatment option compared to medical therapy [13]. Despite this increasing evidence for primary surgical resection of isolated ileitis terminalis, recommendations for operative treatment in CD patients remain controversial throughout different health care systems [14,15]. According to the Guidelines of the American College of Gastroenterology, surgery should be reserved for severe enteric complications such as obstruction, perforation, or abscess formation. In addition, CD refractory to medical treatment is also regarded as an indication for surgical intervention, but with the drawback that operations become increasingly complex and challenging as the state of inflammation worsens [16]. In contrast, individual European societies have a less conservative approach to performing bowel resections in patients with IBD and CD, in particular. The British National Institute for Health and Care Excellence (NICE), for instance, recommend surgery as an alternative to medical treatment at relatively early time points of the disease, providing that the risks and benefits as well as personal preferences of patients are carefully considered [17]. In comparison, German guidelines only recommend surgery for limited symptomatic inflammation to the ileocecal region as an alternative primary therapy. This is due to the fact that the large majority of CD patients requires surgery at least once during their lifetime while surgical resection results in a prolonged disease-free survival (DFS) compared to medical treatment [18].

Despite the increasing evidence that surgery should be considered as a treatment alternative in cases of limited Crohn’s Disease, consensus recommendations remain heterogeneous. Consequently, patients are mostly treated medically after primary diagnosis without introduction to surgical options. This attests to the need for further studies investigating the value of surgery in CD treatment. Thus, the objective of this single-center retrospective study was to investigate and evaluate the feasibility and prognostic outcome of primary ICR in patients with ileocecal CD in comparison to primary anti-inflammatory and immunosuppressive therapy, followed by surgery at a later stage.

## 2. Materials and Methods

### 2.1. Study Population

In this single-center retrospective study, all patients with ileocecal resection due to Crohn’s Disease with terminal inflammatory, penetrating, and/or stricturing ileitis (Montreal classification B1-B3) treated from 2006 to 2017 at the Department of Surgery at the University Hospital of Wuerzburg were evaluated. 169 patients with ICR were identified. Patients with previous gastrointestinal surgery or ulcerative colitis were excluded.

Terminal ileitis was defined as inflammation limited to the terminal ileum with the histopathological diagnosis of Crohn’s Disease. Recommendation about individual treatment regimen was given by an interdisciplinary board including gastroenterologists and surgeons. The preoperative extent of inflammation was usually assessed by endoscopy and completed by an MRI scan. Extraintestinal disease was evaluated by patient history. Postoperative 2-years follow-up assessment was performed by a phone interview with patients including a defined questionnaire about further medical and surgical/endoscopic treatments and complications after index operation (ICR).

All patients were divided into two subgroups. Patients with primary resection following initial diagnosis of terminal ileitis without previous medical therapy were compared to patients initially receiving anti-inflammatory and/or immunosuppressive medication followed by ICR at a later stage. Sociodemographic and clinicopathological data including time of diagnosis, stricture/stenosis, penetrating disease behavior, abscess, and additional disease manifestations were collected for each patient from patient records. Further, surgical procedure as an open or laparoscopic ICR, elective or emergency procedure, and creation of a stoma were evaluated. Surgical and non-surgical complications within 30 days after operation, and length of hospital stay were also analyzed from patient records.

### 2.2. Outcome

The primary endpoint was defined as the need for anti-inflammatory or immunosuppressant therapeutics within two years following surgery. Anti-inflammatory and immunosuppressive therapy included corticosteroids, biologicals, and other medications (aminosalicylate, methotrexate, thiopurine) according to national and international guidelines [18]. Secondary endpoints were the time interval between the start of medications and ICR, a possible escalation of the medical therapy, and the need for an additional surgical procedure due to disease progression. Further secondary endpoints included surgical and non-surgical complications within 30 days and the length of hospital stay.

### 2.3. Subgroup Analysis for Localized Ileocecal Crohn Manifestation

For a subgroup analysis, all patients with localized ileocecal Crohn’s manifestation were included. Patients were analyzed for primary and secondary endpoints, and patients as well as disease characteristics were evaluated.

### 2.4. Statistical Analysis

Descriptive data are presented as median with range or total numbers with percentage. Differences in patient characteristics were assessed by Chi-Square test, Fisher’s exact test, or ANOVA test in accordance to the data scale and distribution. A *p*-value of <0.05 was considered statistically significant. Statistical analysis was performed by using SPSS statistics (Version 25, IBM, Armonk, NY, USA).

### 2.5. Ethical Considerations

Ethical approval for this study was obtained from the Ethics Committee of the University of Wuerzburg, Germany (Reference: 6619-sc/10 July 2019).

## 3. Results

### 3.1. Patient Characteristics

**Figure 1:**
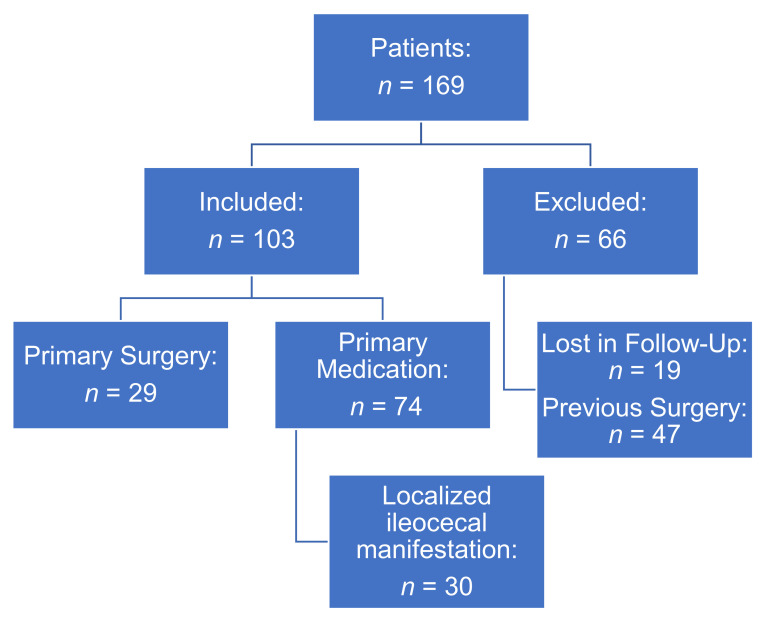
Study design.

In this single-center study, 169 patients with ICR were initially identified. While 66 patients were excluded due to failed follow-up (*n* = 19) and previous surgery (*n* = 47), 103 patients were finally included with 29 patients receiving primary resection and 74 patients treated with medication before surgery (Figure 1). As presented in Table 1, both groups did not show any significant differences regarding age, gender, BMI, and comorbidities (cardiovascular, pulmonary, diabetes). Primary manifestations of CD were strictures/stenosis, penetration, and fistulas with comparable incidences between both subgroups. However, while the majority of patients with primary ICR suffered from a localized disease to the ileocecal area, patients with primary medication showed increased rates of multi-localized disease manifestations in the upper gastrointestinal tract (6.9% versus 20.3%) and perianal (6.9% versus 27.0%, *p* = 0.031). Median time interval for patients with primary surgical intervention from diagnosis to surgical intervention was 15.6 months compared to 85.8 months for patients with primary medication (*p* < 0.001).

### 3.2. Postoperative Outcome

Emergency procedures were significantly increased in the group of patients with primary surgery (17.2% versus 2.7%, *p* = 0.018). On the other hand, patients who were initially treated with anti-inflammatory or immunosuppressant medication had a significantly enhanced rate of open ileocecal resection compared to patients who received primary resection (64.9% versus 34.5%, *p* = 0.007). While more patients with short disease interval received a laparoscopic approach (44.8% versus 29.7%), rates of temporary stomas were comparable between both groups (6.9% versus 5.5%) without any permanent stoma. Furthermore, no significant differences between both groups were seen for postoperative surgical complications. Rates of anastomotic leakage did not differ significantly (6.9% versus 8.1%). Similarly, no differences were seen for intraabdominal abscesses, wound infection and ileus postoperatively between both groups. In addition, non-surgical complications (pneumonia, urinary tract infection/UTI, thrombosis) and length of hospital stay (10 versus 9 days) were comparable in both groups without relevant disparities (Table 2).

### 3.3. Postoperative Immunosuppressive Therapy

Two years after surgery, significantly more patients received anti-inflammatory or immunosuppressive therapy who had been initially treated medically in comparison to patients who were primarily operated (37.9% versus 77.0%, *p* < 0.001) (Table 2). Following surgery, patients with primary medical therapy received significantly more steroids (13.8% versus 35.1%, *p* = 0.05) as well as biologicals (17.2 versus 37.8%, *p* = 0.06) and other anti-inflammatory medication (aminosalicylate, methotrexat, thiopurine) (17.2 versus 32.4%, *p* = 0.09) postoperatively. Furthermore, median time interval between surgery and the start of medical treatment was significantly longer for those who did not receive any medication prior to surgery (14 months versus 1 month, *p* < 0.001). While the need for a second Crohn-associated surgical intervention was low for both groups (3.4% versus 1.4%) during the 2-year follow-up, escalation of immunosuppressive or anti-inflammatory medication within two years past surgery was significantly increased in patients who already received medical treatment prior to surgery in comparison to patients with primary surgical intervention (3.4% versus 27%, *p* = 0.006).

### 3.4. Outcome for Localized Ileocecal Diseases Manifestation

Subgroup analysis was performed for all patients with localized CD to the terminal ileum. While all patients with primary resection had localized disease, 30 patients who received primary medical therapy could be included. Patient characteristics were similar between both groups, and no differences were seen for comorbidities or active smoking. However, patients who received primary resection had significantly more fistulas in comparison to patients who received surgery after primary medical therapy (Table 3). Further, when patients were treated primarily medically and had ICR at a later stage, they received significantly more immunosuppressive medication postoperatively compared to patients with early surgery (80% versus 37.9%, *p* = 0.001) and had a shorter time interval until the start of medical therapy after ICR (1 versus 14 months, *p* < 0.001) despite localized disease manifestation. While rates of therapy escalation were slightly increased in the group of late surgery during the two year follow-up (3.4% versus 16.7%), the increase in immunosuppressive medication was mainly due to steroids (13.8% versus 50%, *p* = 0.005) (Table 3).

## 4. Discussion

Despite its high clinical and socioeconomic relevance due to its increasing incidence and prevalence worldwide, therapeutic options for Crohn’s Disease remain limited without curative perspectives. Patients with CD often have reduced quality of life and increased health care related costs [4,5,19]. Since disease progression can be very heterogenous, individual treatment strategies are necessary to improve these criteria for this complex disease. We showed in our study that patients with primary resection in cases of localized CD needed significantly less anti-inflammatory or immunosuppressive medication postoperatively compared to patients with primary medical treatment several years before surgery was initiated. Therefore, while CD is very heterogenous with varying degrees of disease severity throughout patients and is often primarily treated by medication, surgical resection represents an effective alternative therapy in case of localized disease.

Despite major efforts in basic and clinical research, the etiology of CD is still not understood in detail. Evidence indicates that various factors such as environment, microbiota, and genetics result in decreased epithelial barrier function and an upregulated immune system [20,21,22]. While the precise of events in the pathophysiology of CD remains controversial, most medications currently target the immune system. Besides their undisputed anti-inflammatory properties and clinical efficiency, they can result in serious side effects are inadequate for many patients [3]. Consequently, numerous medications are often prescribed for CD over lengthy time periods which significantly reduce quality of life and incur high health care related costs.

In cases of limited disease, de Groof et al. have shown that laparoscopic ileocecal resection is cost-effective in comparison to anti-inflammatory medication and that quality of life is at least similar between patients with Crohn-related surgery and medical therapy [13]. Similarly, two retrospective studies demonstrated a significantly prolonged disease-free survival for patients receiving primary surgical resection compared to primary medical treatment with reduced need for immunosuppressive therapy [8,23]. Further, studies confirmed the positive effect of early surgical intervention on immunosuppressive medication with a reduced rate of surgical re-operation [24,25,26]. In addition, a single prospective randomized trial demonstrated that laparoscopic ileocecal resection can be an alternative to infliximab therapy in patients not responding to three months of conventional medical therapy (glucocorticoids, thiopurines, methotrexate) [10]. The trial showed improved quality of life for patients after surgery compared to medical treatment without differences in morbidity. In addition, 37% of patients receiving infliximab needed surgical intervention during follow-up, underlining the positive effect of early surgery.

While laparoscopic surgery is usually preferred over conventional open surgery due to fewer complications and fewer incisional hernias [27], both techniques are similar regarding disease recurrence [28,29,30]. Specifically, disease recurrence is significantly decreased after surgical resection in comparison to patients receiving anti-inflammatory or immunosuppressant therapy with more than 50% of patients being relatively or completely symptom-free for the former in the long-term [31,32,33]. This data led to consensus recommendations of different national and international guidelines to consider surgery in case of localized inflammation as an alternative to medical treatment at relatively early disease stages. In particular, British as well as German guidelines propose that patients are informed at early time points about both therapeutic strategies taking into account the state of disease, individual risks and benefits, as well as personal preferences for further treatment recommendations [17,18]. Similarly, updated European Crohn’s and Colitis Organization (ECCO) guidelines recommend primary surgery in cases of localized CD as a reasonable alternative to infliximab therapy [34]. However, despite low morbidity and decreased rates of disease recurrence for Crohn-related surgery, American guidelines consider surgical resection as a treatment option only in case of enteric complications such as bowel obstruction, perforation, abscess formation, or in the presence of medically refractory disease [16]. This perspective is underlined by recent data from Australia and Canada, demonstrating a trend towards decreasing numbers of Crohn-related surgery due to differences in clinical practice [14,35].

Because of these contrasting perspectives on the role of surgery in Crohn’s Disease, we analyzed our patient cohort comparing primary surgical resection to initial medical therapy with anti-inflammatory or immunosuppressant medication followed by surgery in case of terminal ileitis. While most patient characteristics were similar between both groups, patients with primary surgery had significantly more fistulas as a primary manifestation (72.4% versus 47.3%, *p* = 0.021) (Table 1). In cases of primary medical treatment, more patients were initially diagnosed with multilocular disease mainly located in the perianal area as well as in the upper gastrointestinal tract besides the ileocecal area. Accordingly, the feasibility of laparoscopic resection was increased at early time points of disease progression (44.8% versus 29.7%). While advanced disease stage can make a laparoscopic approach impossible due to excessive inflammation and development of fistulas, no differences were seen for the need of a stoma (6.9% versus 5.5%) or postoperative complications. The median time interval until the start of immunosuppressive or anti-inflammatory medication postoperatively was significantly shorter for patients with primary medical therapy (1 month) in comparison to patients with primary surgery as a treatment of choice (14 months, *p* = 0.001). This was in part due to its prophylactic application to maintain remission postoperatively, especially in patients with a long interval of disease progression before surgery and in those with multilocular disease, which were primarily treated medically. However, within two years after surgery, only 37.9% of patients who were primarily operated on at an early stage of CD needed medical therapy compared to 78.4% of patients who received surgical resection following primary medical treatment at a later stage (*p* < 0.001). Patients with primary medical treatment needed significantly more steroids as well as biologicals, and therapy escalation was only necessary for one patient treated by primary surgery (3.4%), while 20 patients (27%) with surgery at a later stage received therapy escalation postoperatively (*p* = 0.006). However, despite the increased need for postoperative medical therapy in patients with surgery at a later disease stage, rates for a second Crohn-associated surgery remained low in both groups.

To further strengthen our conclusion, we next performed a subgroup analysis for patients with localized ileocecal disease and excluded all patients with multilocular manifestations. Despite localized CD to the terminal ileum, patients who were initially treated by medication received statistically significant more immunosuppressive medication after ICR than patients with primary resection (37.9% versus 80.0%, *p* = 0.001) starting immediately after surgery. Consequently, since ICR is effective in treating patients with localized ileocecal disease, automatic postoperative continuation of medication for patients with a long history of medical treatment might be discussed on an individual basis and based on established risk factors (active smoking, fistulating phenotype, etc.) [36]. Additionally, a structured endoscopic surveillance using the Rutgeert score should be performed after 6 months to re-evaluate the therapeutic decision and adjust it if necessary [36]. Moreover, while patients who primarily received ICR had an increased incidence of fistulas, only a small number of those were additionally treated medically postoperatively without the need for therapy escalation during the follow-up period. Interestingly, while fistulas are generally seen as risk factors for disease recurrence and are therefore often treated by additive medication as remission prophylaxis, our data demonstrate that additional therapy after ICR might not be necessary in cases of fistulas.

Our study has some limitations, including its retrospective character and the single-center design. In addition, the follow-up period of 24 months is relatively short and lacking in long-term data on re-operation, functional outcome, and life quality. However, the explicit differentiation between the two groups of primary surgery and primary medication is a major advantage for robust disease-free analysis. Furthermore, while the overall cohort is heterogenous regarding the disease manifestation with 59% of patients with primary medical therapy showing a multilocular disease manifestation, our cohort represents the clinical reality and demonstrates the challenges of CD therapy. However, in our cohort, we focused on patients receiving surgery mainly due to failure of medical treatment, and, thus, patients without surgical intervention benefiting from medical therapy were not part of our study. Additionally, our study was not designed to analyze and compare the therapeutic outcome following surgery and medical treatment in general, but investigating the effect of primary versus late surgery in patients with localized ileocecal CD.

## 5. Conclusions

In conclusion, the optimal treatment regimen for patients with Crohn’s Disease remains controversial. Here, we were able to demonstrate that ICR as a primary treatment for patients with limited ileocecal CD is a safe and effective option at relatively early stages of localized CD, which might result in a reduction of immunosuppressive medication postoperatively. Furthermore, our data might question general paradigms of CD treatment as automatic continuation of preoperative medication postoperatively and additive medical therapy in cases of fistulas. Further studies in basic research to not only inhibit inflammation, but also support mucosal healing as well as clinical studies with larger cohorts and a prospective design are necessary to improve the therapy of CD. Since CD represents a heterogenous and complex disease, interdisciplinary communication between gastroenterologists and surgeons on a regular base is required to provide optimal care for patients.

## Figures and Tables

**Table 1:** Patient demographics and clinical characteristic.

	Primary Surgery (*n* = 29)	Primary Medication (*n* = 74)	*p-*Value
Age, years			
Median	27	29.5	ns ^3^
Range	14–27	13–61	
Gender (*n*, %)			
Male	18 (62.1)	39 (52.7)	ns
Female	11 (37.9)	35 (47.3)	
Body Mass Index (kg/m^2^)			
Median	22.6	21.8	ns
Range	16.5–40.4	13.5–32.7	
ASA classification (*n*, %)			
>2	4 (13.8)	9 (12.2)	ns
Active smoking (*n*, %)	11 (37.9)	19 (25.7)	ns
Comorbidities (*n*, %)			
Cardiovascular	6 (20.7)	21 (28.4)	ns
COPD ^1^/Asthma	2 (6.9)	8 (10.8)	ns
Diabetes mellitus	0	2 (2.7)	ns
Crohn’s manifestation (*n*, %)			
Stricture/Stenosis	20 (69.0)	63 (85.1)	ns
Penetrating	3 (10.3)	5 (6.8)	ns
Fistula	2 (72.4)	35 (47.3)	0.021
Additional localizations of Crohn’s Disease (*n*, %)			
Upper GI ^2^ tract	2 (6.9)	15 (20.3)	ns
Colorectal	13 (44.8)	36 (48.6)	ns
Perianal	2 (6.9)	20 (27.0)	0.031
Extraintestinal	1 (3.4)	10 (13.5)	ns
Weight loss >10% in 12 months prior to surgery (*n*, %)	10 (34.5)	29 (39.7)	ns
Time interval from diagnosis to surgery, months (mean)	15.6	85.8	<0.001

Abbreviations:^1^ chronic obstructive pulmonary disease; ^2^ gastrointestinal; ^3^ not significant.

**Table 2:** Postoperative outcome of all patients.

	Primary Surgery (*n* = 29)	Primary Medication (*n* = 74)	*p*-Value
Surgical priority (*n*, %)			
Elective	24 (82.8)	72 (97.3)	0.018
Emergency	5 (17.2)	2 (2.7)	
Surgical procedure (*n*, %)			
Open	10 (34.5)	48 (64.9)	0.007
Laparoscopic	13 (44.8)	22 (29.7)	
Conversion	6 (20.7)	4 (5.4)	
Stoma (*n*, %)			
Temporary	2 (6.9)	4 (5.5)	ns
Permanent	0	0	
Surgical complications, 30 days (*n*, %)			
CDC ^1^ ≥ 3a	4 (13.8)	9 (12.2)	ns
Anastomotic leak	2 (6.9)	6 (8.1)	
Abscess	2 (6.9)	3 (4.3)	
Wound infection	2 (6.9)	2 (2.7)	
Ileus	0	2 (2.7)	
Non-surgical complications, 30 days (*n*, %)			
Pneumonia	1 (3.4)	0	ns
UTI ^2^	0	4 (5.4)	
Thrombosis	0	1 (1.4)	
Length of hospital stay, days			
Median	10	9	
Range	6–72	6–51	ns
Additive medical therapy 2 years after surgery (*n*, %)	11 (37.9)	58 (78.4)	<0.001
Time until start of additive medical therapy after surgery, months			
Median	14	1	<0.001
Range	1–82	1–54	
Medical therapy escalation 2 years after surgery (*n*, %)	1 (3.4)	20 (27.0)	0.006
Immunosuppressive therapy 2 years after surgery (*n*, %)			
Steroids	4 (13.8)	30 (40.5)	0.05
Biologicals	5 (17.2)	28 (37.8)	0.06
Others	5 (17.2)	24 (32.4)	0.09
2nd Crohn associated operation 2 years after primary surgery (*n*, %)	1 (3.4)	1 (1.4)	ns

^1^ Clavien Dindo classification; ^2^ urinary tract infection.

**Table 3:** Postoperative outcome in patients with localized (ileocecal) manifestation.

	Primary Surgery (*n* = 29)	Primary Medication (*n* = 30)	*p*-Value
Age at diagnosis, years			
Median	26	24	ns
Range	14–72	6–55	
Age at surgery, years			
Median	27	29.5	ns
Range	14–72	13–57	
Crohn’s manifestation (*n*, %)			
Stricture/Stenosis	20 (69.0)	25 (83.3)	ns
Penetrating	3 (10.3)	0	ns
Fistula	21 (72.4)	9 (30.0)	0.002
Perianal	2 (6.9)	0	ns
Extraintestinal	1 (3.4)	0	ns
Surgical complications, 30 days (*n*, %)			
CDC ^1^ ≥ 3a	4 (13.8)	3 (10.0)	
Anastomotic leak	2 (6.9)	2 (6.7)	ns
Abscess	2 (6.9)	1 (3.3)	
Wound infection	2 (6.9)	0	
Ileus	0	0	
Length of hospital stay, days			
Median	10	8	ns
Range	6–72	6–29	
Additive medical therapy 2 years after surgery (*n*, %)	11 (37.9)	24 (80.0)	0.001
Time until start of additive medical therapy after surgery, months			
Median	14	1	<0.001
Range	1–82	1–29	
Medical therapy escalation 2 years after surgery (*n*, %)	1 (3.4)	5 (16.7)	0.195
Immunosuppressive therapy 2 years after surgery (*n*, %)			
Steroids	4 (13.8)	15 (50.0)	0.005
Biologicals	5 (17.2)	9 (30.0)	0.36
Others	5 (17.2)	6 (20.0)	1

^1^ Clavien Dindo classification.

## Data Availability

Institutional database. Therefore, restrictions to availability apply due to data protection regulations. Anonymized data are, however, available from the corresponding author on reasonable request.

## References

[1] Ng S.C., Shi H.Y., Hamidi N., Underwood F.E., Tang W., Benchimol E.I., Panaccione R., Ghosh S., Wu J.C., Chan F.K. (2018). Worldwide incidence and prevalence of inflammatory bowel disease in the 21st century: A systematic review of population-based studies. Lancet.

[2] Kappelman M.D., Rifas-Shiman S.L., Porter C.Q., Ollendorf D.A., Sandler R.S., Galanko J.A., Finkelstein J.A. (2008). Direct health care costs of Crohn’s disease and ulcerative colitis in US children and adults. Gastroenterology.

[3] Hisamatsu T., Matsumoto T., Watanabe K., Nakase H., Motoya S., Yoshimura N., Ishida T., Kato S., Nakagawa T., Esaki M. (2019). Concerns and Side Effects of Azathioprine During Adalimumab Induction and Maintenance Therapy for Japanese Patients With Crohn’s Disease: A Subanalysis of a Prospective Randomised Clinical Trial [DIAMOND Study]. J. Crohns Colitis.

[4] Voskuil M.D., Bangma A., Weersma R.K., Festen E.A.M. (2019). Predicting (side) effects for patients with inflammatory bowel disease: The promise of pharmacogenetics. World J. Gastroenterol..

[5] Pillai N., Dusheiko M., Maillard M.H., Rogler G., Brüngger B., Bähler C., Pittet V.E.H. (2019). The Evolution of Health Care Utilisation and Costs for Inflammatory Bowel Disease Over Ten Years. J. Crohns Colitis.

[6] Burisch J., Kiudelis G., Kupcinskas L., Kievit H.A.L., Andersen K.W., Andersen V., Salupere R., Pedersen N., Kjeldsen J., D’Incà R. (2019). Natural disease course of Crohn’s disease during the first 5 years after diagnosis in a European population-based inception cohort: An Epi-IBD study. Gut.

[7] Wong D.J., Roth E.M., Feuerstein J.D., Poylin V.Y. (2019). Surgery in the age of biologics. Gastroenterol. Rep..

[8] Latella G., Cocco A., Angelucci E., Viscido A., Bacci S., Necozione S., Caprilli R. (2009). Clinical course of Crohn’s disease first diagnosed at surgery for acute abdomen. Dig. Liver Dis..

[9] Golovics P.A., Lakatos L., Nagy A., Pandur T., Szita I., Balogh M., Molnar C., Komaromi E., Lovasz B.D., Mandel M. (2013). Is early limited surgery associated with a more benign disease course in Crohn’s disease?. World J. Gastroenterol..

[10] Ponsioen C.Y., de Groof E.J., Eshuis E.J., Gardenbroek T.J., Bossuyt P.M., Hart A., Warusavitarne J., Buskens C.J., van Bodegraven A.A., Brink M.A. (2017). Laparoscopic ileocaecal resection versus infliximab for terminal ileitis in Crohn’s disease: A randomised controlled, open-label, multicentre trial. Lancet Gastroenterol. Hepatol..

[11] Shimada N., Ohge H., Kono T., Sugitani A., Yano R., Watadani Y., Uemura K., Murakami Y., Sueda T. (2019). Surgical Recurrence at Anastomotic Site After Bowel Resection in Crohn’s Disease: Comparison of Kono-S and End-to-end Anastomosis. J. Gastrointest. Surg..

[12] Flemming S., Kim M., Germer C.T. (2020). Terminal ileitis in Crohn’s disease-Is primary surgery the better treatment?. Chirurg.

[13] de Groof E.J., Stevens T.W., Eshuis E.J., Gardenbroek T.J., Bosmans J.E., van Dongen J.M., Mol B., Buskens C.J., Stokkers P.C.F., Hart A. (2019). Cost-effectiveness of laparoscopic ileocaecal resection versus infliximab treatment of terminal ileitis in Crohn’s disease: The LIR!C Trial. Gut.

[14] Toh J.W.T., Wang N., Young C.J., Rickard M., Keshava A., Stewart P., Kariyawasam V., Leong R. (2018). Major Abdominal and Perianal Surgery in Crohn’s Disease: Long-term Follow-up of Australian Patients With Crohn’s Disease. Dis. Colon Rectum.

[15] Toh J.W., Stewart P., Rickard M.J., Leong R., Wang N., Young C.J. (2016). Indications and surgical options for small bowel, large bowel and perianal Crohn’s disease. World J. Gastroenterol..

[16] Lichtenstein G.R., Loftus E.V., Isaacs K.L., Regueiro M.D., Gerson L.B., Sands B.E. (2018). ACG Clinical Guideline: Management of Crohn’s Disease in Adults. Off. J. Am. Coll. Gastroenterol. ACG.

[17] NICE Guideline (2019). Management of Crohn’s disease. BMJ.

[18] Preiß J.C., Bokemeyer B., Buhr H.J., Dignaß A., Häuser W., Hartmann F., Herrlinger K.R., Kaltz B., Kienle P., Kruis W. (2014). Updated German Clinical Practice Guideline on Diagnosis and Treatment of Crohn’s Disease. Z. Gastroenterol..

[19] Knowles S.R., Graff L.A., Wilding H., Hewitt C., Keefer L., Mikocka-Walus A. (2018). Quality of Life in Inflammatory Bowel Disease: A Systematic Review and Meta-analyses-Part I. Inflamm. Bowel. Dis..

[20] De Souza H.S.P., Fiocchi C., Iliopoulos D. (2017). The IBD interactome: An integrated view of aetiology, pathogenesis and therapy. Nat. Rev. Gastroenterol. Hepatol..

[21] Brazil J.C., Parkos C.A. (2016). Pathobiology of neutrophil-epithelial interactions. Immunol. Rev..

[22] Li N., Shi R.H. (2018). Updated review on immune factors in pathogenesis of Crohn’s disease. World J. Gastroenterol..

[23] Aratari A., Papi C., Leandro G., Viscido A., Capurso L., Caprilli R. (2007). Early versus late surgery for ileo-caecal Crohn’s disease. Aliment. Pharmacol. Ther..

[24] Lee J.M., Lee K.M., Kim J.S., Kim Y.S., Cheon J.H., Ye B.D., Kim Y.H., Han D.S., Lee C.K., Park H.J. (2018). Postoperative course of Crohn disease according to timing of bowel resection: Results from the CONNECT Study. Medicine.

[25] An V., Cohen L., Lawrence M., Thomas M., Andrews J., Moore J. (2016). Early surgery in Crohn’s disease a benefit in selected cases. World J. Gastrointest. Surg..

[26] Riss S., Schuster I., Papay P., Herbst F., Mittlbock M., Chitsabesan P., Stift A. (2014). Surgical recurrence after primary ileocolic resection for Crohn’s disease. Tech. Coloproctol..

[27] Patel S.V., Patel S.V., Ramagopalan S.V., Ott M.C. (2013). Laparoscopic surgery for Crohn’s disease: A meta-analysis of perioperative complications and long term outcomes compared with open surgery. BMC Surg..

[28] Dasari B.V., McKay D., Gardiner K. (2011). Laparoscopic versus Open surgery for small bowel Crohn’s disease. Cochrane Database Syst. Rev..

[29] Eshuis E.J., Polle S.W., Slors J.F., Hommes D.W., Sprangers M.A., Gouma D.J., Bemelman W.A. (2008). Long-term surgical recurrence, morbidity, quality of life, and body image of laparoscopic-assisted vs. open ileocolic resection for Crohn’s disease: A comparative study. Dis. Colon Rectum.

[30] Tan J.J., Tjandra J.J. (2007). Laparoscopic surgery for Crohn’s disease: A meta-analysis. Dis. Colon Rectum.

[31] Eshuis E.J., Slors J.F., Stokkers P.C., Sprangers M.A., Ubbink D.T., Cuesta M.A., Pierik E.G., Bemelman W.A. (2010). Long-term outcomes following laparoscopically assisted versus open ileocolic resection for Crohn’s disease. Br. J. Surg..

[32] Lowney J.K., Dietz D.W., Birnbaum E.H., Kodner I.J., Mutch M.G., Fleshman J.W. (2006). Is there any difference in recurrence rates in laparoscopic ileocolic resection for Crohn’s disease compared with conventional surgery? A long-term, follow-up study. Dis. Colon Rectum.

[33] Coffey C.J., Kiernan M.G., Sahebally S.M., Jarrar A., Burke J.P., Kiely P.A., Shen B., Waldron D., Peirce C., Moloney M. (2018). Inclusion of the Mesentery in Ileocolic Resection for Crohn’s Disease is Associated With Reduced Surgical Recurrence. J. Crohns Colitis.

[34] Adamina M., Bonovas S., Raine T., Spinelli A., Warusavitarne J., Armuzzi A., Bachmann O., Bager P., Biancone L., Bokemeyer B. (2020). ECCO Guidelines on Therapeutics in Crohn’s Disease: Surgical Treatment. J. Crohns Colitis.

[35] Dittrich A.E., Sutton R.T., Haynes K., Wang H., Fedorak R.N., Kroeker K.I. (2020). Incidence Rates for Surgery in Crohn’s Disease Have Decreased: A Population-based Time-trend Analysis. Inflamm. Bowel. Dis..

[36] Peyrin-Biroulet L., Bouhnik Y., Roblin X., Bonnaud G., Hagege H., Hebuterne X. (2017). French national consensus clinical guidelines for the management of Crohn’s disease. Dig. Liver Dis..

